# Heart failure in type 2 diabetes: current perspectives on screening, diagnosis and management

**DOI:** 10.1186/s12933-021-01408-1

**Published:** 2021-11-06

**Authors:** Antonio Ceriello, Doina Catrinoiu, Chanchal Chandramouli, Francesco Cosentino, Annique Cornelia Dombrowsky, Baruch Itzhak, Nebojsa Malić Lalic, Francesco Prattichizzo, Oliver Schnell, Petar M. Seferović, Paul Valensi, Eberhard Standl, the D&CVD EASD Study Group

**Affiliations:** 1 IRCCS MultiMedica, Via Gaudenzio Fantoli, 16/15, 20138 Milan, Italy; 2 Faculty of Medicine, Clinical Center of Diabetes, Nutrition and Metabolic Diseases, Ovidius University of Constanta, Constanta, Romania; 3 Duke-NUS Medical School, Singapore, Singapore; 4 National Heart Research Institute, National Heart Centre, Singapore, Singapore; 5 Unit of Cardiology, Karolinska Institute, Karolinska University Hospital Solna, Stockholm, Sweden; 6 Sciarc GmbH, Baierbrunn, Germany; 7 Clalit Health Services and Technion Faculty of Medicine, Haifa, Israel; 8 School of Medicine, Clinic for Endocrinology, Diabetes and Metabolic Diseases, University of Belgrade, Belgrade, Serbia; 9 Forschergruppe Diabetes e. V. at Helmholtz Centre Munich GmbH, Munich, Germany; 10 School of Medicine, University of Belgrade, Belgrade University Medical Center, Belgrade, Serbia; 11 Unit of Endocrinology, Diabetology, Nutrition, Jean Verdier Hospital, AP-HP, CRNH-IdF, CINFO, Paris 13 University, Bondy, France

**Keywords:** Type 2 diabetes, Heart failure, NT-proBNP, Guidelines

## Abstract

Type 2 diabetes is one of the most relevant risk factors for heart failure, the prevalence of which is increasing worldwide. The aim of the review is to highlight the current perspectives of the pathophysiology of heart failure as it pertains to type 2 diabetes. This review summarizes the proposed mechanistic bases, explaining the myocardial damage induced by diabetes-related stressors and other risk factors, i.e., cardiomyopathy in type 2 diabetes. We highlight the complex pathology of individuals with type 2 diabetes, including the relationship with chronic kidney disease, metabolic alterations, and heart failure. We also discuss the current criteria used for heart failure diagnosis and the gold standard screening tools for individuals with type 2 diabetes. Currently approved pharmacological therapies with primary use in type 2 diabetes and heart failure, and the treatment-guiding role of NT-proBNP are also presented. Finally, the influence of the presence of type 2 diabetes as well as heart failure on COVID-19 severity is briefly discussed.

## Introduction

Heart failure (HF) is a heterogeneous disease with an age-related increase in prevalence, from 1% at the age of 50–59 years to 10% at 75 years and older [1]. The increasing prevalence of HF in the elderly population may be attributed to the elevated number of long-term survivors after myocardial infarction (MI) who are particularly prone to develop left ventricular dysfunction (LVD) [1], a main driver of HF [2]. In addition to age, obesity and diabetes have been identified as important risk factors for HF [2]. HF often manifests as the first cardiovascular (CV) event in people with type 2 diabetes (T2D) [3]. Even individuals with pre-diabetes, as defined by the criteria of the World Health Organization (WHO) and the American Diabetes Association (ADA), are at a 9–58% greater risk of developing HF [4]. It is also to be noted that individuals with HF and pre-diabetes have a higher risk of all-cause mortality and cardiac outcomes compared to those with normoglycemia [5]. In general, clinically manifest HF is present in 10–30% of all subjects with T2D, especially common at the age of 70 years and older, while 30–40% of all cases of acute or chronic HF have prevalent T2D [6]. Individuals with established T2D have a 33% greater risk for hospitalization for HF (HHF) than individuals without T2D [7]. The prevalence of unrecognized HF in those with T2D is thought to be considerable [6]. For all these reasons, the recent Universal Definition and Classification of HF recognized T2D as a prime risk factor for incident HF, suggesting individuals with T2D as being in the first stage of HF (stage A) [8]. In addition, HF itself is emerging as an antecedent for T2D development, as suggested by prospective cohort studies [9]. Thus, T2D and HF are interrelated: T2D increases the risk of HF, HF is highly prevalent in patients with T2D, and HF might increase the risk of developing T2D [10].

In the overall adult population of developed countries, the prevalence of clinically detectable HF is estimated to be 1–2% [2]. In contrast to this registry-based prevalence, a meta-analysis based on echocardiographic screening studies of the general population showed a prevalence rate estimate of 4.2%, suggesting a potentially high number of undetected cases of HF in the general population [2]. However, while the incidence of HF is increasing in people older than 85 years but also at younger age in recent years [11] and the epidemiologic linkage between T2D and HF is clear [6], recognition of those at risk and/or those with prevalent HF is lagging. This is because the diagnosis of HF is challenging, which may be attributable to misclassification of HF as chronic obstructive pulmonary disease, deconditioning, aging, and unavailability of screening tools in primary care setting [2]. Additionally, a major challenge in diagnosing HF is the asymptomatic presentation of this disease at early stages [12]. The similarity in symptoms between HF and other conditions such as obesity further fuels the risk of under-/misdiagnosis [2]. A large observational study with a median follow-up of 37.7 months showed that the cumulative incidence of CV death or HHF among subjects with HF and T2D (~ 43%) was almost double compared with patients with HF without diabetes (~26%) [13]. The overall survival rates after diagnosing HF have been reported to be 24.5% at 10 years with a decreasing rate from 64.7% in 45–54-year-old patients to 4.4% in 85- to 94-year-old patients [14].

In this review, we describe the pathophysiology of the different HF subtypes, focusing on the myocardial alterations induced by T2D in the absence of coronary disease and other risk factors, i.e., cardiomyopathy in diabetes. We synthesize the complex interplay among the heart, the kidney, and the metabolism in diabetes. We emphasize the current criteria used for HF diagnosis and the gold standard screening tools for individuals with T2D. Then, we discuss the current evidence on pharmacological therapies with primary use in T2D and HF and the treatment-guiding role of NT-proBNP. Finally, we highlight the influence of the presence of T2D as well as HF on COVID-19 severity.

## Classification of heart failure and epidemiological aspects

The diagnosis of HF had been traditionally characterized by groups based on left ventricle ejection fraction (LVEF). A recent ‘Report on the Universal Definition and Classification of Heart Failure’ [8] and the 2021 European Society of Cardiology (ESC) guidelines [15] suggested three main categories of HF as defined by the ejection fraction (EF): HF with preserved EF (HFpEF, LVEF ≥ 50%), HF with mildly reduced EF (HFmrEF, LVEF between 41 and 49%) and HF with reduced EF (HFrEF, LVEF ≤ 40%) .

HFrEF accounts for about half of all HF cases with a trend of decreasing prevalence from 1987 to 2001 [16] and a decreasing incidence from 1990 to 2009 in the United States [17] which contrasts with the overall increase in HF incidence [11]. Risk factors specific for HFrEF include male sex and a history of CV diseases (CVD) such as MI [18]. The mortality rate of HFrEF patients is slightly higher than for those with HFpEF [16, 19] and is mainly caused by CV death (76% in men, 70% in women) [20]. Similarly, HFpEF accounts for approximately 50% among all HF patients with an increasing prevalence over a 15-year period from 1986 to 2002, making HFpEF the predominant form of HF in the future [16, 21]. As a third and distinct HF subtype, HFmrEF was introduced in 2013 by the American College of Cardiology Foundation/American Heart Association (ACCF/AHA) [22], and in 2016 by the ESC [18]. Based on retrospective analysis from randomized controlled trials in HFrEF and HFpEF, HFmrEF was recently renamed from “HF with mid-range EF” to “HF with mildly reduced EF” since HFmrEF people benefit from similar therapies to those with HFrEF [15]. However, HFmrEF accounts for 10–25% within the overall HF population [23]. The CHARM study and a meta-analysis demonstrated that the all-cause mortality risk of the HFmrEF group resembles more the HFpEF group, but also opposite data exists here [24, 25].

In general, significant predictors for HF are hypertension, chronic kidney disease (CKD), obesity, and diabetes [26, 27].

### Pathophysiological aspects

There is a change in the framework of our understanding of the pathophysiology of HF from the hemodynamic model to the hypothesis that HF is a progressive LVD which usually results from an index event [28]. LVD can be classified into abnormalities of systolic function or abnormalities of myocardial relaxation, previously known as diastolic dysfunction. The initial step of the clinical manifestation of systolic dysfunction is an injury to myocytes [2, 29], e.g., due to MI or ischemia [2, 30]. Injury-induced myocyte damage and thus progressive myocyte loss provokes an inflammatory response [26] and thereby causes ventricular remodeling [26, 29]. Remodeling generates an imbalanced heart wall structure with eccentric hypertrophy characterized by an increased length of myocytes [26]. The inflammatory process triggers an excessive production of fibrotic tissues, further disturbing cardiac function by an impaired transduction of myocyte contraction into cardiac force, culminating in an uncoordinated contraction of the myocyte bundles [26]. In many cases, HFrEF manifests as systolic dysfunction alone [18, 31]. However, one in every four females and one in every six males with HFrEF also demonstrated abnormalities in myocardial relaxation [18, 31]. Incomplete or slow return of myofibrils in their resting length results in steeper pressure/volume relationships in the left ventricle (LV) with resultant impaired filling of the LV and raised left atrial pressures [32]. This situation is most often caused by increased LV stiffness [26] due to concentric hypertrophy of myocytes causing LV wall thickening [26] and often invoked as a cause of HFpEF [30, 33]. However, when patients with a “normal” LVEF, as measured using 2-dimensional echocardiography, are evaluated with more sensitive means to assess myocardial function such as strain imaging, it is common to detect abnormalities in systolic function not otherwise detectable with conventional imaging techniques [34].

### Cardiomyopathy in diabetes

Cardiomyopathy in people with diabetes (CMiPD), also known as “diabetic cardiomyopathy”, is defined by a cardiac dysfunction due to a suppressed glucose metabolism and elevated fatty acid (FA) metabolism [35], and by the existence of an abnormal myocardial structure and performance in individuals with diabetes who do not show any symptoms/signs of coronary artery disease, valvular disease, and other CV risk factors such as hypertension and dyslipidemia [36]. The pathogenesis of CMiPD can be directly attributed to biological abnormalities in diabetes such as hyperglycemia, hyperinsulinemia, systemic insulin resistance, and low-grade inflammation [36]. Insulin therapy could also be associated with an increased mortality risk than oral hypoglycemic agents irrespective of the LVEF or HF etiology [37]. Therefore, the prevalence of CMiPD is similarly increasing as this of T2D [36]. Of note, the relevance of CMiPD is further supported by a large observational study showing an increased risk of HHF in patients with T2D but no other risk factor [38].

**Fig. 1:**
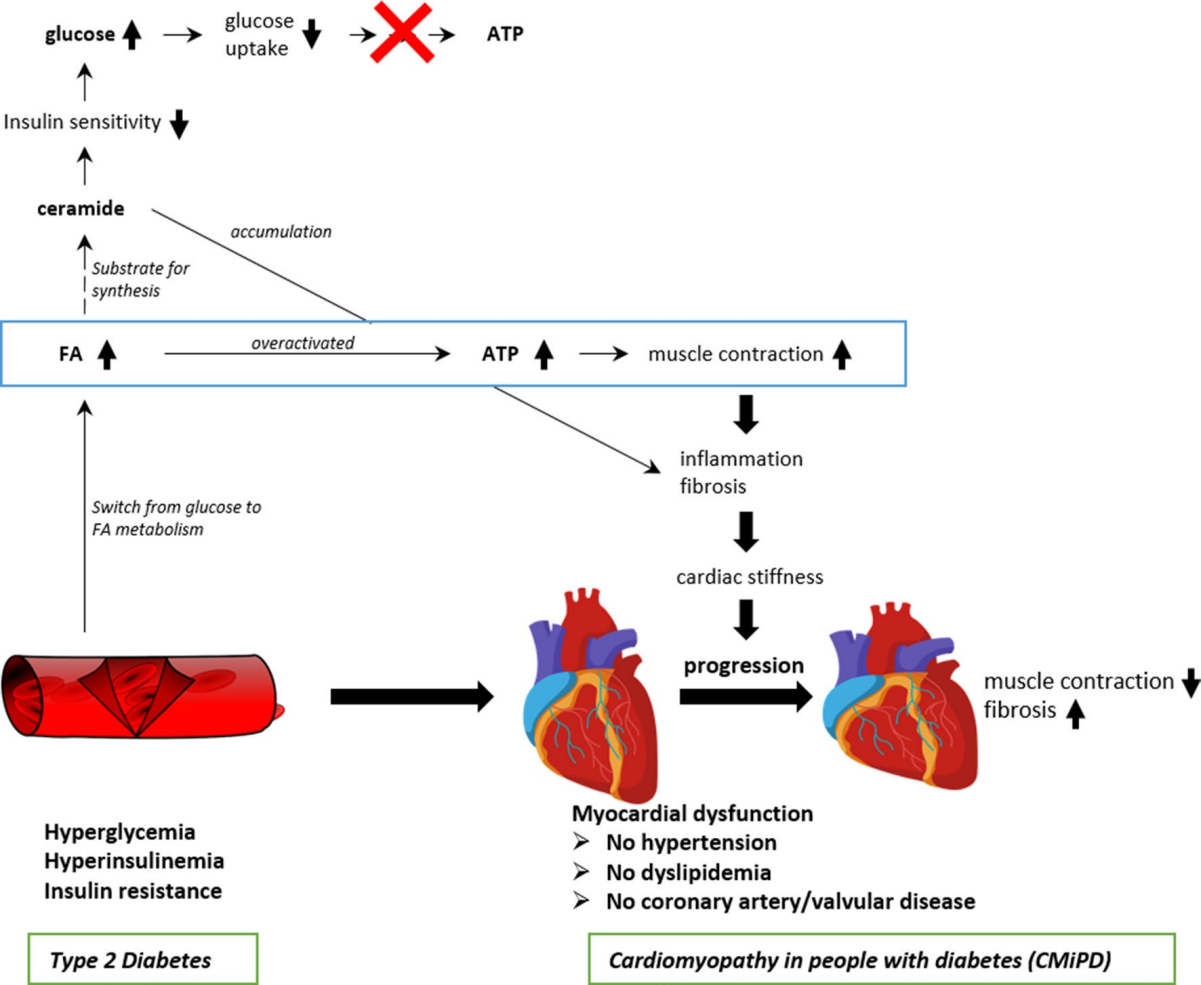
Pathophysiological mechanisms of cardiomyopathy in type 2 diabetes (CMiPD). Fatty acids (FAs) are preferentially metabolized to ATP, sustaining muscle contraction. During progression to severe stages of cardiomyopathy, both the increased FA metabolism and accumulated ceramide contribute to inflammation and fibrosis, leading to cardiac stiffness and reduced contractility

CMiPD can be classified in 4 stages. During progression from stage I, with impaired myocardial relaxation but normal EF, to the final stage IV, with a clinical overt ischemia and infarct causing HF, muscle contraction decreases and fibrosis develops [35]. In addition and parallel to structural changes of the heart, diabetes-associated conditions such as hyperglycemia, hyperinsulinemia, inflammation, and hyperlipidemia can alter cardiac function (Fig. 1) [39]. In CMiPD, the heart muscle shows an impaired glucose metabolism due to insulin resistance, characterized by a reduced glucose uptake, a reduced glycolytic activity, and a reduced pyruvate oxidation [35]. Thus, glucose is limitedly available and there is an overabundance of circulating FAs, another principal fuel of energy next to glucose facilitating ATP production necessary for cardiac contraction, which is mainly consumed by cardiomyocytes in the case of CMiPD [35]. The resulting metabolic inflexibility and the overactive FA oxidation promote a number of secondary pathways which render the heart less able to cope with increasing workloads [35]. In particular, the FA-rich cardiomyocytes produce ATP less efficiently, accumulate lipids and a range of toxic intermediates, which are considered to promote pro-inflammatory and profibrotic responses, finally contributing to hypertrophy and diastolic dysfunction in CMiPD [35]. For example, ceramide are toxic lipids, which are synthesized when an excess of FAs is present [40, 41]. An overload of ceramide accumulates in cardiomyocytes and has profound effects on cellular signaling, such as apoptosis and insulin resistance [41], and facilitates ventricular modeling, fibrosis, and macrophage infiltration upon myocardial infarction [40].

A further factor possibly contributing to the development of CMiPD is mitochondrial dysfunction. In this regard, excessive mitophagy can lead to an imbalance between mitophagy and mitochondrial biogenesis and may thus result in an exacerbated destruction of myocardial cells [42]. However, further investigations are necessary to clarify if mitophagy plays differing roles in the pathophysiology of cardiomyopathy in type 1 diabetes (T1D) and T2D [42].

#### Interplay between metabolic disturbances and the heart

**Fig. 2:**
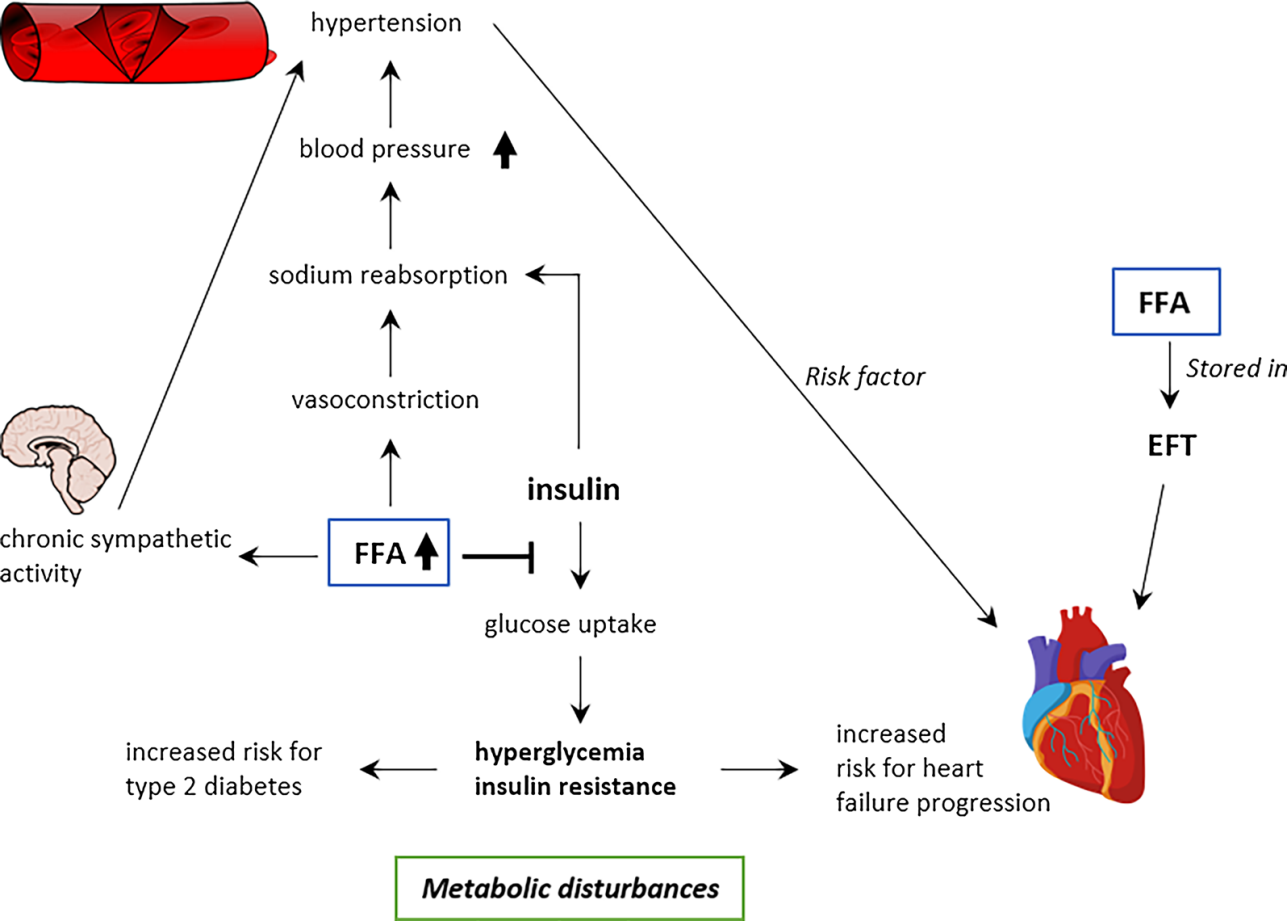
Mechanism for interaction between the metabolism disturbances and the heart. Insulin interferes with the free fatty acids (FFAs) metabolism. Consequently, a high amount of FFAs is present in the bloodstream, which impairs the insulin-mediated glucose uptake leading to hyperglycemia and insulin resistance, and promotes hypertension via increased sodium reabsorption and chronic sympathetic activity. In addition, insulin itself promotes hypertension. Metabolic disturbances impart a high risk for type 2 diabetes and heart failure progression, apart from FFA-rich epicardial fat tissue (EFT) that also facilitates heart failure

An important risk factor for the development of T2D and HF are metabolic disturbances, a pathological condition encompassing a range of insulin resistance-driven metabolic alterations [43, 44]. High circulating levels of free FA (FFA) are particularly evident in subjects with metabolic disturbances [45], which results from an altered FFA metabolism [45, 46]. Mechanistically, there is an impaired insulin-mediated downregulation of lipolysis in adipocytes of subjects with metabolic disorders, which leads to an increased amount of FFAs in the bloodstream (Fig. 2) [45]. FFAs abolish insulin’s action in muscle and liver, and consequently induce hyperglycemia and insulin resistance which is considered to increase the T2D risk [46]. Furthermore, the link between metabolic disturbances, T2D, and HF is based on the presence of insulin resistance which is closely linked to hypertension [46], a prominent risk factor for HF [26, 27]. Also, hyperinsulinemia and an excess of FFAs contribute to hypertension [46, 47] by mediating an increased sodium retention in the kidney [46] and by enhancing sympathetic activity chronically [48]. Moreover, the vasodilative effect of insulin is abolished in case of insulin resistance, and hyperinsulinemia increases renal sodium reabsorption, thereby promoting hypertension [48]. In addition, epicardial fat tissue (EFT) is thought to facilitate the development of CVD, in particular HF [49]. Indeed, the excess of FFAs might be removed and accumulated by the EFT [49], which is distributed along coronary arteries between pericardium and myocardium [50]. In a setting of chronic, low-grade systemic inflammation, the amount of EFT is elevated, and its role potentially changes from a heart- protecting and supporting structure to a local source of pro-inflammatory cytokines in the heart, which probably worsens endothelial dysfunction and leads to increased cardiac fibrosis and stiffness and thus inducing HF, in particular HFpEF [51]. The amount of EFT is significantly higher in people with diabetes than in those without diabetes [50], possibly providing an additional mechanistic link between diabetes and HF development [43].

#### Interplay between the heart and the kidney

**Fig. 3:**
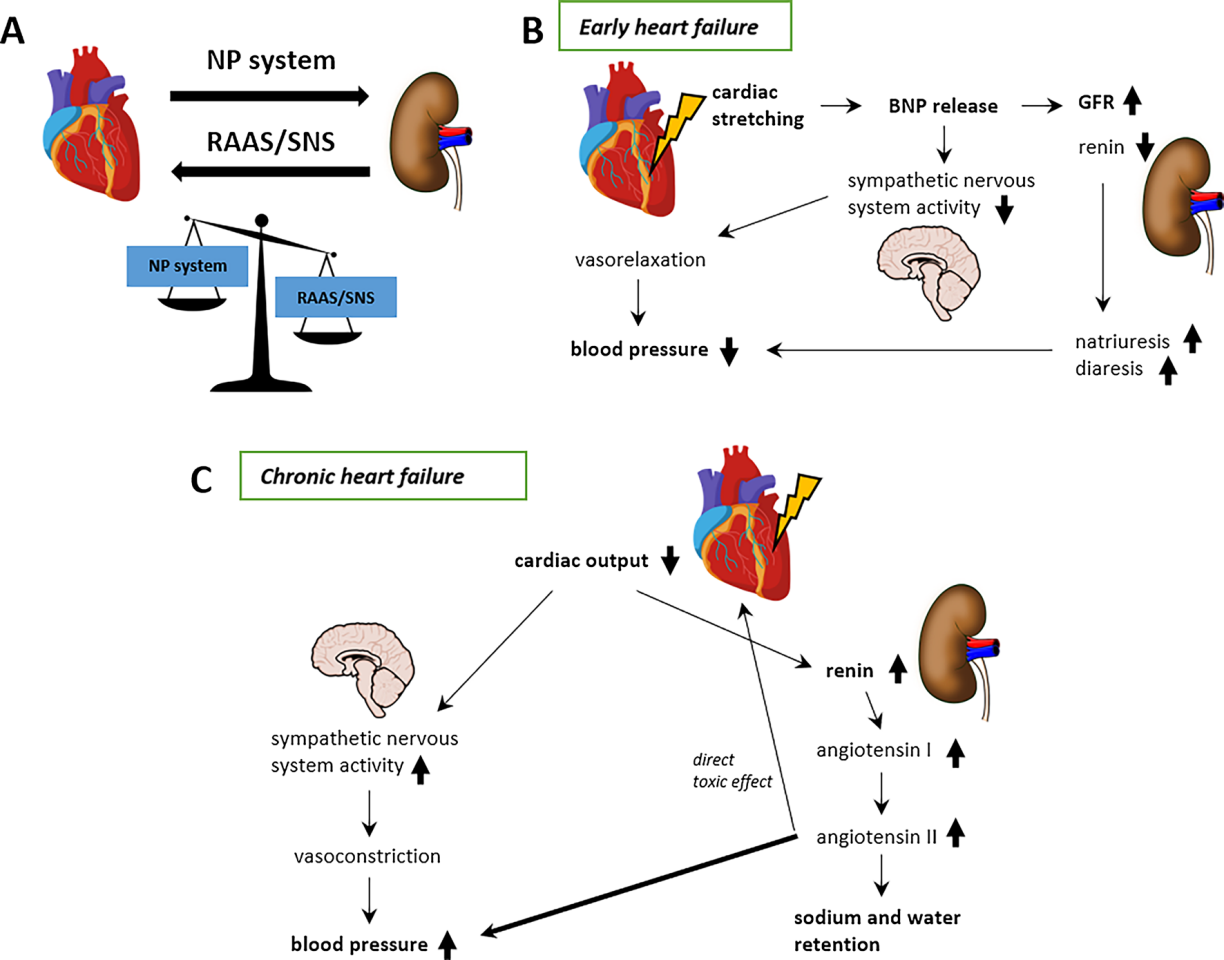
Heart-kidney interaction is mediated by the natriuretic peptide system counterbalanced by the RAAS and the SNS. In early heart failure, the natriuretic peptide action dominates, leading to suppression of the SNS manifested as vasorelaxation and to natriuresis and diuresis in the kidney, which all translate into a lowered blood pressure. In chronic heart failure, the effectiveness of natriuretic peptides is lost, and actions of the overactivated RAAS and SNS dominate leading to vasoconstriction and expression of renin and angiotensin, which both mediate an increased blood pressure

In diabetes, both HF and renal dysfunction are frequently co-existent [52]. About one-third of subjects with chronic HF suffer from diabetes, of whom about the half also have prevalent CKD [53]. This inter-relationship results from a bidirectional communication between both organs via neurohumoral processes (Fig. 3). Myocyte damage itself can overactivate several systemic neurohumoral pathways [29]. Both the sympathetic nervous system (SNS) and the renin–angiotensin–aldosterone-system (RAAS) activate key pathways in both HF and CKD [54], which are counteracted by the natriuretic peptide system [55]. There are several markers for assessing renal function in people with diabetes, which are linked to HF. Microalbuminuria, also a marker for vascular dysfunction, is highly prevalent in HF subjects without [56] and with diabetes, having a prevalence between 10% in the general population and 20–30% in T2D individuals [57, 58]. The presence of microalbuminuria in people with T2D doubles the risk for a CV event, making it a widely recognized, strong, and independent marker of increased CV risk and all-cause mortality [58, 59]. A second marker for renal function assessment is the estimated glomerular filtration rate (eGFR). Reduction in eGFR has been shown to be both highly prevalent and longitudinally predictive of the development of HF in people with T1D and T2D, and eGFR reductions are a stronger predictor of unfavorable outcomes in HF [60]. For subjects with T1D, the HHF risk was two times greater at eGFR 45–60 mL/min/1.73 m^2^ and ≥ three times greater at eGFR < 30 mL/min/1.73 m^2^ when compared to normal eGFR [61]. Similar to T1D, several CV outcome trials observed an approximately 2-fold increase of the HHF rate in people with T2D and a reduced eGFR [62]. Furthermore, an observational study analyzing people with T2D and new-onset HF showed a decrease in survival with increasing eGFR [63]. Specifically, survival decreased from 2.8 years at an eGFR of 45–59 mL/min/1.73 m^2^ to 0.7 years at an eGFR of < 15 mL/min/1.73 m^2^ [63]. In general, the hazard ratio for CV events is estimated to be 2.2 for each halving of the baseline eGFR in T2D subjects [64].

### Biomarkers and glycemic markers for HF and diabetes

#### Glycemic markers for diabetes in people with HF

Diabetes is diagnosed by repetitive assessment of the glycemic parameters HbA1c or fasting plasma glucose (FPG) (level of evidence: B) in the 2019 ESC-EASD recommendations) [10]. The 2006/2011 World Health Organization and 2019 American Diabetes Association (ADA) recommendations defined the presence of diabetes when HbA1c is ≥ 6.5% (48 mmol/mol) and FPG is ≥7.0 mmol/L (126 mg/dL) [10]. Moreover, performing an oral glucose tolerance test (OGTT) is a well recommended tool since it detects postprandial hyperglycemia, a risk factor of CVD, and thus identifies a large number of undiagnosed patients with diabetes [65, 66].

It is well known that worse glycemic control, i.e., higher levels of HbA1c, is associated with worsening cardiac structure and function [67]. In this regard, Lee and colleagues conducted a prospective study including patients with acute HF with and without diabetes from the Korean Acute Heart Failure Registry (KorAHF). The results showed that an insufficient glycemic control (HbA1c ≥ 7.0% within one year after discharge) was significantly associated with a higher risk for all-cause mortality compared with an adequately controlled diabetes (HbA1c < 7.0%) [68]. However, the type of effect glycemic control has on HF outcomes depends on the medication used to lower HbA1c. Lee and colleagues also examined in a further study the effect of insulin therapy on HF outcomes in patients with diabetes (KorAHF registry). An increase in mortality was observed in patients with good glycemic control treated with insulin (HbA1c < 7.0%) compared to patients who received oral hypoglycemic agents, indicating a possible negative effect of insulin therapy [37]. Subclinical damage of the heart increases linearly across the glycemic spectrum from no diabetes over pre-diabetes to diabetes [69]. Moreover, the presence of subclinical myocardial damage increases the risk for CV events and HF, with the highest CV risk for people with T2D [69]. Pre-diabetes and undiagnosed diabetes increase the risk of HHF, CV, and all-cause death [70] and worsen the prognosis of people with HFrEF [4, 5, 70]. However, it is critical to note that diagnosing dysglycemia does not prevent HFrEF subjects from adverse clinical outcomes [70]. Beyond glycemic control, visit-to-visit HbA1c variability was also reported to predict the occurrence of HF [71], and recent evidence suggested that glucose variability is an emerging risk factor for CVD in diabetes [72].

#### Natriuretic peptides

In recent years, the circulating biomarkers of the natriuretic peptide system have become increasingly important in relation to HF and, when elevated in the absence of prevalent HF, identify individuals with “pre-HF” at risk for progression to symptomatic disease [8]. Cardiac stress induces the synthesis of a 108 amino acid long precursor peptide pro-BNP in myocytes [73] which is split into a biologically active BNP and an inactive N-terminal peptide, NT-proBNP [73]. Both BNP and NT-proBNP are viewed as useful biomarkers to support clinical judgement for the diagnosis of HF [8, 73]. In addition, NT-proBNP correlates with the HF risk in people with T2D [74] and with adverse outcomes in HF subjects [75].

Both NT-proBNP and BNP have a predictive value for both short- and intermediate-term CV events in individuals with diabetes [76, 77]. Indeed, the SAVOR-TIMI 53 trial reported that diabetic individuals without known CVD but with elevated NT-proBNP levels have a 3-fold increased risk of HF than counterparts with known CVD and normal NT-proBNP levels [78]. Similarly, the ADVANCE trial and other studies demonstrated that NT-proBNP strongly predicts HF risk, the overall excess mortality, and CV mortality in people with T2D [74, 79]. In the PONTIAC trial, high-risk people were T2D pre-selected based on NT-proBNP levels showing a beneficial effect in terms of primary prevention of hospitalization and death due to cardiac events [80], and the STOP-HF trial supports the finding that BNPs are a reliable screening tool in an at-risk population in reducing newly-diagnosed HF and the prevalence of LV dysfunction [81]. NT-proBNP levels also correlate significantly with the functional NYHA classes of HF [82]. However, despite their importance as prognostic tests, natriuretic peptides are still not used regularly in ambulatory care settings.

#### Troponin

Besides NT-proBNP, cardiac troponin T (cTnT) is one of the most established cardiac biomarkers, used as the gold standard to detect myocardial injury [83]. As part of the contractile apparatus of the heart, it is released into the bloodstream when myocyte’s membrane disrupts after cardiomyocyte injury [84]. Detection of cardiomyocyte injury, particularly when identified using a high sensitivity assay (e.g., hs-cTnT), has an important role for prognosticating HF onset, hospitalization [85, 86], and all-cause and CV mortality [87] of patients with diabetes [85]. Mechanistically, the presence of chronic hyperglycemia has been associated with myocardial injury, reflected by higher concentrations of hs-cTnT [86] as well as rising hs-cTnT over time [88]. These findings suggest that abnormalities in the various pathways discussed above may directly lead to cardiomyocyte injury, setting up a potential risk for the development of CMiPD. Moreover, HF patients with diabetes can only be distinguished from those without diabetes by TnT values but not by NT-proBNP values [85]. However, the TnT-derived prognosis is similar for HFrEF people with and without diabetes despite a greater prevalence of elevated TnT levels in individuals with diabetes [85]. In general, the prognostic value of troponin in T2D individuals is difficult to interpret since cut-off values have not been uniformly defined and are always specific for the used assay [83].

In addition to the listed biomarkers NT-proBNP and cTnT, it is essential to indicate that patients with acute HF and diabetes may have a different biomarker profile than patients with acute HF and without diabetes. In this context, a network analysis performed by Sharma and colleagues revealed that patients with acute HF and diabetes have significantly different levels of various biomarkers than patients without diabetes [89]. To mention are, among others, markers for inflammation (TNFR-1a, periostin), cardiomyocyte stretch (BNP), and angiogenesis (VGEFR, angiogenin), suggesting that cardiac remodeling, inflammation, and fibrosis are closely associated with each other in patients with HF and diabetes [89, 90].

## Detection of heart failure in diabetes

### *Diagnosing HF*

The Heart Failure Society of America, the Heart Failure Association of the ESC, the Japanese Heart Failure Society and the Writing Committee of the Universal Definition of Heart Failure agreed on a uniform definition and classification of HF in 2021 [8]. According to this universal definition, HF is a clinical syndrome with current or prior symptoms and/or signs (Table 1), which are caused by structural and/or functional abnormalities of the heart [8]. The presumption of HF is to be confirmed by at least one of the following examinations:

- Elevated natriuretic peptide levels.
- Objective evidence of cardiogenic pulmonary or systemic congestions by diagnostic modalities or hemodynamic measurements [8].

As mentioned earlier, HF is mainly categorized in HFrEF, HFpEF, and HFmrEF on the basis of LVEF. However, it is critical to emphasize that over-dependence on LVEF alone to define HF is fraught with risk for misdiagnosis, as substantial inter-reader variation exists in the assessment of LVEF, and a significant percentage of about 50% [90] of patients with severe HF have normal LVEF.

The progression of HF can be assessed by different grading schemes (Table 1). The new universal definition proposed a HF staging in “at risk”, “pre-HF”, “HF”, and “advanced HF” that enhances clinician, patient, and public understanding and adoption as well as takes into account the evolving role of circulating biomarkers to define patients with structural and subclinical heart disease who are at high risk for developing HF and are candidates for targeted HF prevention treatments strategies [8]. Furthermore, the New York Heart Association (NYHA) functional classification categorizes HF in stage I to IV, focusing on exercise intolerance and symptom severity [18]. There is also the A/B/C/D classification of the 2009 ACCF/AHA guidelines with a focus on HF development and progression [91].

**Table 1:** Comparison of the HF staging defined by different international guidelines

Proposed New Staging of HF according to the universal definition [8]		Updates guidelines of the ACCF/AHA in 2009 [91]		NYHA functional classification [92]	
At risk (Stage A)	At risk for HF, but without current or prior symptoms/signs of HF and without structural cardiac changes or elevated biomarkers	A	At high risk for HF but without structural heart disease or symptoms of HF	None	–
Pre-HF (Stage B)	Without current or prior symptoms/signs of HF with evidence of one of the following: ¬ Structural Heart Disease ¬ Abnormal cardiac function ¬ Elevated natriuretic peptide levels or troponin levels	B	Structural heart disease but without signs or symptoms of HF	I	No limitation of physical activity. Ordinary physical activity does not cause symptoms of HF.
HF (Stage C)	Current or prior symptoms and/or signs of HF caused by a structural and/or functional cardiac abnormality	C	Structural heart disease with prior or current symptoms of HF		
				II	Slight limitation of physical activity. Comfortable at rest, but ordinary physical activity results in symptoms of HF.
				III	Marked limitation of physical activity. Comfortable at rest, but less than ordinary activity causes symptoms of HF.
Advanced HF (Stage D)	Severe symptoms and/ or signs of HF at rest, recurrent hospitalizations despite GDMT, refractory or intolerant to GDMT, requiring advanced therapies	D	Refractory HF requiring specialized interventions	IV	Unable to carry on any physical activity without symptoms of HF, or symptoms of HF at rest.

### Monitoring of clinical symptoms and signs

Several clinical symptoms and signs listed in Table 2, are similar for patients with HFrEF, HFmrEF, and HFpEF and need to be assessed at every visit [18]. Both symptoms and signs can be used for monitoring treatment response and its stability over time [18]. However, there are difficulties in accurately diagnosing HF solely based on symptoms and signs since both are often non-specific, especially in the early stages of the disease, or they are masked by other conditions, especially in elderly individuals with other comorbid conditions [18]. Moreover, some signs indicate specifically HF, but they are hard to detect in clinical practice and are poorly reproducible [18]. In general, diagnosing HFpEF is more challenging than HFrEF. This is caused by the absence of a dilated LV in HFpEF patients who have an increased LV wall thickness instead and/or an increased left atrial size as a sign of an enhanced filling pressure [18].

**Table 2:** Overview of symptoms and signs of HF according to the universal definition of HF [8]

Symptoms		Signs	
Typical	Less typical	More specific	Less specific
• Breathlessness • Orthopnea • Paroxysmal nocturnal dyspnea • Reduced exercise tolerance • Fatigue • Tiredness • Ankle swelling • Inability to exercise • Swelling of parts of the body other than ankles • Bendopnea	• Nocturnal cough • Wheezing • Bloated feeling • Postprandial satiety • Loss of appetite • Decline in cognitive function • Confusion (especially in the elderly) • Depression • Dizziness • Syncope	• Elevated jugular venous pressure • Third heart sound (gallop rhythm) • Summation gallop with third and fourth heart sounds • Cardiomegaly • Laterally displaced apical impulse • Hepatojugular reflux • Cheyne Stokes respiration in advanced HF	• Unintentional weight gain (>2 kg/week) • Weight loss (in advanced HF) with muscle wasting and cachexia • Tissue wasting (cachexia) • Cardiac murmur • Peripheral oedema (ankle, sacral, scrotal) • Pulmonary rales • Reduced air entry and dullness to percussion at lung bases suggestive of pleural effusion • Tachycardia • Irregular pulse • Tachypnoea • Hepatomegaly • Ascites • Cold extremities • Oliguria • Narrow pulse pressure

### Recommendations for screening and diagnosing heart failure in people with diabetes

1. Electrocardiography (ECG):
  ECG is recommended by the 2021 ESC guidelines [15] as one measure to assess people with suspected acute or chronic HF and present symptoms and/or signs. In people with acute HF, ECG is recommended at the time of admission, during hospitalization, and pre-discharge [15]. If the ECG is abnormal, the likelihood of HF is increasing [15]. ECG allows additionally screening for unrecognized MI and thus could be helpful for risk stratification among high-risk persons for cardiovascular disease [93]. Furthermore, it provides information about etiology (e.g., MI, AF) and guides therapy [15]. Nevertheless, the main use of an ECG is to rule out HF since a completely normal ECG indicates that HF is unlikely [15]. The 2019 ESC guidelines on diabetes developed in collaboration with the European Society for the Study of Diabetes (EASD) (ESC-EASD recommendations) recommend a resting ECG in general when patients with diabetes have been diagnosed with hypertension [10].
2. Echocardiography:
  The clinical diagnosis of remodeling in HF is based on the detection of morphological changes such as changes in the cavity diameter, mass (hypertrophy and atrophy), and geometry (heart wall thickness and shape) [94]. Echocardiography is the most useful, non-invasive diagnostic method to detect these structural changes [94] for evaluating systolic and diastolic dysfunction [95]. Therefore, the 2021 ESC guidelines [15] and the 2019 ESC-EASD recommendations [10] define echocardiography as the first-choice approach to (1) assess cardiac function (LVEF and other parameters) for people with chronic HF [15] and (2) to evaluate structural and functional abnormalities in people with diabetes since increased LV mass (LVM) and diastolic dysfunction are widely found in asymptomatic people [10]. Also, elevated LVM is common in cases of hypertension [67]. It is also well known that an increase of the LVM is already present with increasing age, obesity, and dyslipidemia, all common risk factors for T2D [67], but it also depends on body size and gender [96]. Also, LV hypertrophy is a frequent abnormality in asymptomatic people with T2D [97]. It was found in one-third of those without hypertension, even after excluding silent coronary disease [97]. The LVM indexed to bovine serum albumin (BSA) allows the definition of reference values for the comparison of subjects with different body sizes [96]. Normal values for LVM/BSA defined by the American Society for Endocrinology range from 43 to 95 g/m^2^ for women and 49–115 g/m^2^ for men [96].
3. Biomarker screening:
  The 2021 ESC guidelines (level of evidence: B) [15] and the updated guidelines of the American College of Cardiology/American Heart Association/Heart Failure Society of America (ACC/AHA/HFSA) in 2017 [98] recommend measurement of natriuretic peptides (either NT-proBNP or BNP) to identify “pre-HF” among individuals with diabetes. In contrast, the 2019 ESC-EASD guidelines do not recommend routine assessment of circulating biomarkers in general for CV risk stratification of asymptomatic patients with diabetes [10]. However, the 2021 ESC guidelines and the 2017 ACC/AHA/HFSA guidelines argue that risk stratification by natriuretic peptide measurement can help to identify people at risk of developing HF (ACC/AHA stages A or B) who require further cardiac investigation by a cardiologist and initiate an early intervention for preventing HF [15, 98]. For acute HF people, determination of natriuretic peptides is recommended at the time of admission and pre-discharge [15]. Similarly, the universal definition of HF [8] defines the HF stages by elevated levels of natriuretic peptides and therefore recommends assessing NT-proBNP or BNP routinely in patients without current or prior symptoms or signs of HF. The universal definition of HF suggested the cut-off values for BNP and NT-proBNP of 35 pg/mL and 125 pg/mL for ambulatory HF people and of 100 pg/mL and 300 pg/mL for hospitalized/decompensated HF people, respectively [8]. However, diagnosis of HF cannot be made solely on the basis of natriuretic peptide values because their diagnostic accuracy is influenced by CV and non-CV causes that weaken the informative value of the natriuretic peptide under conditions of obesity, AF, increasing age, and kidney disease [15]. Therefore, natriuretic peptide determination is recommended for ruling out HF but not for diagnosing HF [15]. To complement the informative diagnostic value of natriuretic peptides, further novel biomarkers are needed, including, e.g., secreted Frizzled-related proteins as independent biomarkers for myocardial fibrosis or risk stratification in HF [99–102] or the gut microbiota-derived trimethylamine N-oxide (TMAO) [103].
4. Assessment of glycemic parameters in HF subjects:
  Undiagnosed dysglycemia in people with HFrEF imparts a particularly poor prognosis [70]. Therefore, the 2019 ESC-EASD guidelines recommend the determination of HbA1c and FPG to screen for diabetes in people with pre-existing CVD, with an OGTT carried out if FPG and HbA1c are inconclusive (level of evidence: A) [10]; and the 2021 ESC guidelines recommend routine blood tests including fasting glucose and HbA1c in patients with suspected chronic HF to screen for treatable causes of HF and co-morbidities which affect HF (level of evidence: C) [15].
5. Strategies in people with diabetes to reduce the risk for HF:
  The 2019 ESC-EASD guidelines recommend routine assessment of microalbuminuria and eGFR in order to identify patients at high risk of renal dysfunction or future CVD [10]. In addition, the Standards of Care 2021 from the ADA [104], the 2017 ACC/AHA/HFSA guidelines [98], and the 2019 ESC-EASD guidelines [10] recommend a blood pressure target of <130/80 mmHg (but not < 120 mmHg) in individuals with diabetes at CV risk since hypertension control is associated with a lower HF risk. The 2021 ESC guidelines agree with this recommendation but without target recommendations [15]. In this context, it should be noted that masked hypertension in which ambulatory or home BP but not office BP readings are in the hypertensive range [105] is highly prevalent in patients with T2D [106], making out-of-office BP monitoring a reasonable screening measure for this clinical condition [107]. Patients with diabetes and hypertension should be examined by ECG at resting state in order to detect silent MI, which occurs in 4 % of all diabetic patients, as insult for HF [10]. Also, lifestyle changes and the administration of RAAS blockers as first-line treatment for blood pressure control are recommended for pre-diabetic people and the treatment of hypertensive people with diabetes [10]. Moreover, the administration of RAAS blockers lowers the risk of new-onset diabetes and reduces the risk of sudden cardiac death in HFrEF subjects [10].

In addition to hypertension, an increased body mass index is regarded as a risk factor for HF. Therefore, the 2021 ESC guidelines recommend that obesity should be managed in order to prevent or delay HF onset [15]. All recommendations from international guidelines for people with diabetes at risk for developing HF are summarized in Table 3.

**Table 3:** Recommendations from international guidelines for people with diabetes who are at risk for developing HF

Recommendations for people with diabetes and at risk for developing HF
• Lower blood pressure to <130/80 mmHg
• Examination by ECG at resting stage
• Routine assessment of microalbuminuria
• Routine assessment of eGFR
• Lifestyle changes
• RAAS blocker administration
• Assessment of body mass index

### Therapeutic considerations of heart failure in diabetes and natriuretic peptide monitoring

The 2021 ESC guidelines [15] recommend treatment of HFrEF and HFmrEF with a combination therapy of angiotensin-converting-enzyme inhibitors/angiotensin II receptor blockers (ACE-I/ARB), angiotensin-receptor-neprilysin-inhibitors (ARNI), beta-blockers, mineralocorticoid-receptor antagonists (MRA), and sodium-glucose cotransporter (SGLT)-2 inhibitors. In contrast to HFrEF and HFmrEF, there is currently no therapy for HFpEF subjects [15, 30]. Hence, HFpEF therapy aims only to improve symptoms and well-being [15, 18] and treatment of comorbidities [15]. However, first evidence of improved outcomes in HFpEF subjects comes from the recently published EMPEROR-preserved trial [108].

1. ACE-I and ARB:
  One important study analyzing the effect of the ACE-I enalapril was the SOLVD trial showing that enalapril is able to reduce the occurrence of diabetes in HF subjects compared to placebo [109]. In order to reduce the HF risk in diabetes, the 2019 ESC-EASD recommendations support the inclusion of an ACE-I or an ARB for blood pressure control in diabetic patients who are intolerant to ACE-I, particularly in the presence of microalbuminuria, albuminuria, proteinuria, or LV hypertrophy [10].
2. ARNI:
  The long-term blood pressure-lowering effect of sacubitril-valsartan (LCZ696), a dual-acting ARNI, was tested in the PARADIGM-HF trial, demonstrating that sacubitril-valsartan can significantly reduce the risk of both death and HHF in individuals with HFrEF in comparison to the ACE-I enalapril [110]. However, in contrast to HFrEF, the PARAGON-HF trial provided hints that treatment with sacubitril-valsartan did not lower the rate of total CV death and HFF among people with HFpEF compared to valsartan alone [111]. This effect was independent of a history of diabetes in patients with HFpEF [111]. Also, the beneficial effect of sacubitril-valsartan in HHF risk reduction was comparable between all participants of the PARADIGM-HF trial with HFrEF and an HbA1c ranging from 5.4 to 8.4% [112]. Moreover, sacubitril-valsartan is superior to enalapril in reducing HbA1c values and lowering the rate of initiation of insulin therapy over three years in patients with both diabetes and HFrEF [113]. Therefore, sacubitril-valsartan is supposed to improve glycemic control in those patients [113].
  The risk reduction by sacubitril-valsartan therapy can be testified by a significant reduction of NT-proBNP levels as shown in the HFpEF population of the PARADIGM-HF trial [114]. This effect was gender independent since sacubitril-valsartan lowered NT-proBNP levels similarly in men and women among the PARAGON-HF cohort having HFpEF and 50% of them suffering from diabetes [115].
  Adverse effects of sacubitril-valsartan therapy observed in the PARADIGM-HF trial [110] and in the PARAGON-HF trial [111] were an increased prevalence of symptomatic hypertension and angioedema, but this was still lower than for dual inhibition of both ACE and neprilysin, especially for angioedema [110]. With regard to this data, the ESC-EASD recommendations on diabetes in 2019 recommend the treatment of HF patients with diabetes who remain symptomatic with sacubitril-valsartan instead of ACE-I [10].
3. MRA
  A meta-analysis showed that MRA treatment improved the clinical outcome in patients with HF and diabetes compared to non-MRA therapy [116]. In detail, spironolactone or eplerenone were able to reduce the all-cause mortality, CV mortality, and HHF in this cohort [116]. Finerenone, a novel non-steroidal MRA, reduced the incidence of death from any cause, CV-related hospitalization or emergency in subjects with HFrEF, CKD and/or diabetes in comparison to eplerenone (ARTS-HF trial) [117]. This treatment effect can be monitored by measuring NT-proBNP as shown by the ARTS-HF trial since finerenone was as effective as eplerenone in reducing the NT-proBNP level by at least 30 % [117].
  Adverse events in the ARTS-HF and other MRA studies included in the meta-analysis mentioned above showed that MRA therapy imparts an increased risk for hyperkalemia [116, 117]. In general, the 2019 ESC-EASD recommendations [10] recommend that people with both HFrEF and diabetes should be treated with MRAs when they remain symptomatic despite treatment with ACE-I or beta-blockers (level of evidence: A). MRAs and sacubitril-valsartan are recommended to reduce the risk of sudden cardiac death in patients with HFrEF and diabetes [10].
4. Metformin
  Metformin monotherapy is the first-line treatment for patients with T2D without CVD and at moderate CV risk according to the 2019 ESC-EASD guidelines on diabetes, and it is recommended for diabetes treatment in patients with HF if the eGFR is stable and ≥30 mL/min/1.73m^2^ (level of evidence: C) [10]. Despite not disappearing concerns about the contraindication of metformin in patients with renal or heart failure due to the perceived risk of lactic acidosis [118], several studies showed that the event rate of lactic acidosis is low in people with T2D and one contraindication such as HF or renal impairment [118]. Therefore, the ADA Standards of Care in 2021 [104] support the 2019 ESC-EASD guidelines [10] that metformin therapy should be continued in individuals with T2D and stable HF if the eGFR remains >30 mL/min/1.73 m^2^, and stopped in unstable or hospitalized patients with HF [104]. Reasons for these recommendations are that (1) metformin therapy of people with T2D and HF reduces total mortality and the risk of death or HHF [119], (2) it delays the development of HF in diabetic people in comparison to sulfonylurea monotherapy [119], and (3) metformin therapy is associated with a decreased risk of CV events and death in diabetic people with a clinical CV risk defined by an NT-proBNP level higher than 300 pg/mL [120]. This finding is confirmed by a multivariate analysis of people with T2D, indicating that metformin treatment is a negative predictor of elevated NT-proBNP levels [121]. Moreover, metformin possesses a cardio-protective effect: Metformin therapy of subjects with T2D and NYHA stages III and IV HF with a mean LVEF of 24 ± 7% at baseline improved LVEF significantly to 30 ± 10% compared to the non-metformin group with 27 ± 9% [122].
5. SGLT-2 inhibitor:
  The therapeutic benefit of SGLT-2 inhibitors on CV outcomes in subjects with T2D and established HF was proven in several clinical trials showing a general cardio-protective effect regardless of the glycemic status [123]. One evidence comes from the DAPA-HF trial demonstrating that dapagliflozin lowered the risk of progressing HF (HHF) and CV-related death in HFrEF people (NYHA class II–IV) independent of the glycemic status [124] and gender [125]. In addition, the EMPEROR-preserved trial provided the first evidence of a cardio-protective effect of empagliflozin on the combined risk of HHF and CV death in subjects with HFpEF, an effect that is independent of the presence of diabetes [108]. In other studies for empagliflozin, a lowered risk of CV death and HHF was also shown in the EMPA-REG OUTCOME trial in people with T2D and a history of CVD [126] and in the EMPEROR-Reduced trial in people with HFrEF regardless of the presence of diabetes [127]. In VERTIS CV, ertugliflozin was non-inferior to placebo with respect to its key secondary outcome of CV death or HHF in subjects with T2D and atherosclerotic CVD, but the trial results did not meet the criteria for superiority (HR = 0.88, 95% CI 0.75–1.03) [128]. There was, however, a 30% reduction in the risk of HHF alone, consistent with the effects of the other SGLT-2 inhibitors on this outcome [129]. In VERTIS CV, a pre-specified analysis also showed that the subgroups of patients with the greatest reduction of HF-related events were those with an eGFR < 60 mL/min/1.73 m^2^ and those with albuminuria [130]. Moreover, emerging evidence suggests for sotagliflozin from the SOLOIST-WHF trial that the dual inhibition of both SGLT-1 and SGLT-2 among subjects with T2D may reduce deaths from CV causes, hospitalizations, and urgent visits for either HFpEF and HFrEF [131]. Compared to placebo, sotagliflozin prevented CV death, HHF, and urgent visits for HF in people with T2D and recent worsening HF when sotagliflozin therapy was initiated before or shortly after discharge [131].
  Not only in people with T2D and established CVD do SGLT-2 inhibitors exhibit a cardio-protective effect, but also in people at high risk for CV events. In the CANVAS trial, canagliflozin reduced the risk of CV-related events to a greater extent than placebo in people with both T2D and concomitant increased CV risk [132]. In addition, the DECLARE-TIMI 58 trial demonstrated that dapagliflozin treatment of individuals with T2D who had or are at risk for atherosclerotic CVD results in a lower rate of HHF and CV-related death [133].
  Considering clinical outcome trials, NT-proBNPs have a predictive value for CV events and mortality. The reduction of CV-related events in people with T2D and CV risk can be attributed in parts to the lowered NT-proBNP concentration in the canagliflozin arm of the CANVAS trial [134]. Also, a trend towards lower NT-proBNP levels in the subgroup with a lower LV diastolic function was observed in the canagliflozin treated group compared to the glimepiride treated arm, as shown in a sub-analysis of the CANDLE trial [135]. In line with canagliflozin, dapagliflozin also lowered NT-proBNP levels significantly more than placebo in the DAPA-HF cohort [124]. Similarly, seven days after randomization, empagliflozin significantly reduced NT-proBNP levels when administered as add-on therapy for T2D people admitted for acute decompensated HF compared with the group treated conventionally with glucose-lowering agents [136]. At the time of writing this publication, there was no data for sotagliflozin on reducing NT-proBNP concentration. However, some evidence arises from another dual SGLT-1/2 inhibitor, licogliflozin, that lowered NT-proBNP in patients with both T2D and HF compared to placebo 12 weeks after randomization [137].
  Due to the class effect of SGLT-2 inhibitors, the ESC-EASD guidelines on diabetes in 2019 recommend the SGLT-2 inhibitors empagliflozin, canagliflozin, and dapagliflozin to lower the risk of HHF in patients with diabetes (level of evidence: A) [10]. Apart from that, the 2021 ESC guidelines recommend ertugliflozin and sotagliflozin in patients with T2D at high risk of CV events to reduce HHF, major adverse CV events (MACE), end-stage renal disease, and CV death as well as sotagliflozin in patients with T2D and HFrEF to reduce HHF and CV death [15]. The 2019 ADA/EASD consensus recommends SGLT-2 inhibitors on top of metformin in people with diabetes and HF (especially HFrEF) in order to reduce HHF, MACE, and CV death [138].
6. Dipeptidyl peptidase 4 inhibitor (DPP4i)
  The DPP4i are a not-superior example for therapy of diabetes in HF patients compared to placebo. The SAVOR-TIMI 53 trial provided hints that the DPP4i saxagliptin worsens the prognosis of people with T2D and history or risk of CV events by increasing the number of people who were hospitalized for HF compared to placebo [139]. However, the risk for HHF correlated with the concentration of NT-proBNP, previous HF, and CKD [139]. Also, the EXAMINE trial [140] and the TECOS trial [141] demonstrated that alogliptin and sitagliptin are statistically robustly non-inferior to placebo with regard to the risk of HF outcome of subjects with T2D and an established CVD, and with no effect on 4-point-MACE [140–142]. In addition, alogliptin was able to reduce NT-proBNP concentration, but this effect was similar in both the placebo arm and the DPP4i arm [140]. In contrast, saxagliptin increased NT-proBNP levels in a similar way as placebo does, but the increase was slightly flatter than for placebo [139]. In general, two meta-analyses investigating the CV safety of DPP4is showed that the effect of DPP4i on risk for HF in people with T2D is uncertain, and there is only weak evidence for an increased HF risk due to a short follow-up and a low quality of evidence of the considered randomized and observational studies [143, 144]. The 2019 ESC-EASD guidelines on diabetes recommend DPP4i administration only when HbA1c targets are not reached after therapy with SGLT-2 inhibitors, metformin and/or GLP-1 receptor agonists [10]. However, sitagliptin and linagliptin can be considered for diabetes treatment in patients with HF due to their neutral effect on HHF risk, while saxagliptin is not recommended for diabetes treatment in patients at risk of HF due to its increased risk for HHF (level of evidence: B) [10]. In addition to saxagliptin, there is no treatment recommendation for pioglitazone for diabetes management in people at risk for HF because it increases the incidence of HF in T2D patients compared with placebo [10, 145].
7. Glucagon-like peptide 1 receptor agonist (GLP-1-RAs)
  The effects of GLP-1-RAs on HF in subjects with diabetes remain controversial. The administration of liraglutide in people with T2D at high CV risk was able to significantly lower the rate of the first occurrence of death from CV causes, nonfatal MI, or nonfatal stroke compared to placebo in the LEADER trial [146]. However, liraglutide was non-superior to placebo with regard to HHF among patients with both T2D and high CV risk [146]. In contrast to the LEADER trial, the FIGHT study [147] examined the effect of liraglutide in individuals with established HFrEF who were recently hospitalized. It showed a trend toward worse cardiac outcomes in the diabetic subgroup after liraglutide administration (HR = 1.54, p = 0.07), while there was no significant difference in the number of deaths or re-hospitalizations for HF in individuals without diabetes between the liraglutide group and the placebo group (HR = 1.02, p = 0.94) [147]. Also, the LIVE trial demonstrated that liraglutide did not change the LVEF, compared to placebo, in HF people with and without diabetes [148]. However, in the LIVE study, a significantly increased number of serious cardiac events was seen in 10% of people with chronic HF treated with liraglutide compared with 3% of people in the placebo group, which raises concerns about the administration of this drug in HFrEF subjects [148]. A neutral effect of GLP-1-RAs on HHF compared with placebo was observed for lixisenatide in the ELIXA trial among subjects with T2D who had a recent acute coronary event [149]. In line with the ELIXA trial, no difference in HHF was demonstrated after the use of semaglutide among patients with T2D who were at high CV risk (SUSTAIN-6 trial) [150]. Regarding natriuretic peptide monitoring, the FIGHT study showed that changes in the NT-proBNP level remained almost constant and did not differ between the liraglutide arm and the placebo arm over a time period of 180 days [147]. However, the presence of beneficial effects of GLP-1-RAs on CV outcomes in people with T2D was identified by a meta-analysis of ELIXA, LEADER, SUSTAIN-6, and other trials showing a 9% reduction of the risk of HFF [151]. However, due to missing safety data and the modest benefit of GLP-1-RAs over placebo on HHF, none of the cardiology societies of Europe and the US recommend treatment of T2D individuals with GLP-1-RAs in order to reduce the HF risk [15, 18, 22, 98].

## Diabetes, HF and COVID-19

Coronavirus (SARS-CoV-2) infected subjects were second most likely to suffer from diabetes mellitus (33.9%) as comorbidity, with arterial hypertension being the most common (73.8%) [152]. In addition to the increased risk of infection, SARS-CoV-2-infected individuals with diabetes are also likely to have serious complications or die, especially in conjunction with CV risk factors such as hypertension, obesity and smoking, especially those who are at poor metabolic control [153]. As serious complications in SARS-CoV-2-infected diabetic people, acute myocarditis (36.6% vs. 15.5%), acute HF (25.3% vs. 5.6%), and acute MI (9.9% vs. 1.4%) have been observed compared to non-diabetics. This is probably associated with the initiation of a severe immune response after infection with SARS-CoV-2 [154]. If diabetes and SARS-CoV-2 infection are accompanied by the risk factors male sex, a longer duration of diabetes, as well as a history of micro- and macrovascular complications and HF, significantly increased death rates have been detected [155]. Irrespective of the presence of diabetes in SARS-CoV-2 infected subjects, a history of HF alone was also significantly associated with the need for hospitalization for coronavirus disease 2019 (COVID-19) and a higher mortality [156]. There was even a remarkably increased prevalence of new-onset HF in healthy, hospitalized patients with COVID-19 [156]. In general, patients with a severe COVID-19 progression exhibit elevated levels of cardiac biomarkers compared to non-severe patients. Increased concentrations of NT-BNP, troponin I, C-reactive protein and interleukin-6 were found to be associated with the increased severity of COVID-19, which emphasizes the increased risk of acute cardiac injury with more severe viral infection [157]. There are general recommendations given by Ceriello et al. [153] on treatment continuation when a SARS-CoV-2 subject is hospitalized in an intensive care unit for COVID-19. If a patient has at least one risk factor (diabetes, hypertension, obesity, smoking) and concomitant HF, MI, CKD, or other CV/kidney disorder, oral antidiabetic drugs and/or subcutaneous insulin administration should be stopped immediately; intravenous insulin administration should be initiated, and treatment with ACE-i/ARBs and statins should be continued [153]. Taken together, diabetes and HF occurring either alone or together are important risk factors for a poor progression and prognosis of the COVID-19 [153]. Moreover, diabetic but also healthy people have a high chance to experience HF events when suffering from COVID-19 [154, 156]. The implementation of telemedicine technology at a time of a global pandemic could be beneficial for better prevention and treatment of HF risk factors such as hypertension [158].

## Conclusions

Heart failure remains an important issue for life expectancy, especially for people with diabetes. New therapeutic tools, such as the SGLT-2 inhibitors, are offering the opportunity for better management of this very serious complication of T2D. Using biomarkers such as NT-proBNP can help diagnose HF early to predict prognosis and therapeutic efficacy of medications for HF or/and diabetes. Therefore, NT-proBNP measurement should be implemented early in the monitoring of subjects with diabetes at high CV risk. The importance of screening for HF in diabetes becomes significant, especially during the COVID-19 pandemic in which people with diabetes and concomitant HF are particularly vulnerable to SARS-CoV-2 infection and often have worse disease progression and prognosis than healthy individuals.

## Data Availability

Not applicable.

## Abbreviations

ACC/AHA/HFSA: American College of Cardiology/American Heart Association/Heart Failure Society of America
ACCF/AHA: American College of Cardiology Foundation/American Heart Association
ACE-I: Angiotensin-converting-enzyme inhibitors
ADA: American Diabetes Association
AF: Atrial fibrillation
ARB: Angiotensin II receptor blockers
ARNI: Angiotensin-receptor-neprilysin-inhibitors
BNP: Brain natriuretic peptide
BSA: Bovine serum albumin
CKD: Chronic kidney disease
CMiPD: Cardiomyopathy in people with diabetes
COVID-19: Coronavirus disease 2019
CV: Cardiovascular
CVD: Cardiovascular disease
cTnT: Cardiac troponin T
DPP4i: Dipeptidyl peptidase 4 inhibitor
EASD: European Association for the Study of Diabetes
ECG: Electrocardiography
EF: Ejection fraction
EFT: Epicardial fat tissue
eGFR: Estimated glomerular filtration rate
ESC: European Society of Cardiology
FA: Fatty acids
FFA: Free fatty acid
GLP-1-RA: Glucagon-like peptide 1 receptor agonist
HbA1c: Hemoglobin A1c
HF: Heart failure
HFmrEF: Heart failure with mildly reduced ejection fraction
HFpEF: Heart failure with preserved ejection fraction
HFrEF: Heart failure with reduced ejection fraction
hs-cTNT: High sensitivity cardiac troponin T
FPG: Fasting plasma glucose
HHF: Hospitalization for heart failure
HR: Hazard ratio
LV: Left ventricle
LVD: Left ventricle dysfunction
LVEF: Left ventricle ejection fraction
LVM: Left ventricle mass
MACE: Major adverse cardiovascular events
MI: Myocardial infarct
MRA: Mineralocorticoid-receptor antagonists
NT-proBNP: N-terminal pro-B-type natriuretic peptide
NYHA: New York Heart Association
OGTT: Oral glucose tolerance test
RAAS: The renin-angiotensin-aldosterone-system (RAAS)
SGLT-2: Sodium-glucose cotransporter 2
SARS-CoV-2: Severe acute respiratory syndrome coronavirus 2
SNS: Sympathetic nervous system
T1D: Type 1 diabetes
T2D: Type 2 diabetes
